# Writing the worlds of genomic medicine: experiences of using participatory-writing to understand life with rare conditions

**DOI:** 10.1136/medhum-2021-012346

**Published:** 2022-04-13

**Authors:** Richard Gorman, Bobbie Farsides

**Affiliations:** Clinical and Experimental Medicine, Brighton and Sussex Medical School, Brighton, Brighton and Hove, UK

**Keywords:** genetics, social science, patient narratives, narrative ethics, medical humanities

## Abstract

The diagnostic and treatment possibilities made possible by the development and subsequent mainstreaming of clinical genomics services have the potential to profoundly change the experiences of families affected by rare genetic conditions. Understanding the potentials of genomic medicine requires that we consider the perspectives of those who engage with such services; there are substantial social implications involved. There are increasing calls to think more creatively, and draw on more participatory approaches, in evoking rich accounts of lived experience. In this article, we discuss our rationale for, and experiences of, using ‘participatory-writing’ to understand the diverse, variable and multilayered everyday lives of families and how these correspond with the emerging, rapidly changing and complex field of genomic medicine. Participatory-writing has many benefits as a method for social inquiry. Writing can be expressive and self-revelatory, providing insight into personal and sensitive topics. Writing together produces new conversations and relationships. Pieces written by participants have the potential to affect readers, evoking and enlivening research and prompting professional change. Working with a writing tutor, we organised a participatory-writing programme for families touched by genetic conditions. This involved a series of workshops with an emphasis on building confidence in expressing lived experience through experimenting with different writing techniques. Afterwards we arranged reflective interviews with participants. We drew on dialogical narrative analysis to engage with participants’ written pieces, and highlight what everyday life is like for the people who live with, and care for, those with genetic conditions. The stories produced through our writing-groups unfold the implications of new genomic technologies, illuminating how genomics acts to (and likewise, fails to) reconfigure aspects of people’s lives outside of the clinic, while simultaneously existing as a sociotechnical frame that can eclipse the wider contexts, challenges and liveliness of life with rare genetic conditions.

## Introduction

Tracing the implications of developments within genetic science has become a major area of research and debate within medical sociology and allied disciplines (Martin and Dingwall 2010). Sociologists and bioethicists have long argued that technological developments are leading to an increasing ‘geneticisation’ of many aspects of health and healthcare (Lippman 1991). This can particularly be seen in the establishment and adoption of a ‘genomics agenda’ within public health institutions (Mwale and Farsides 2021). Genomics involves the study of all of a person’s genes (the genome) and how genes interact with each other and the environment. The expansion of this area of medicine has been made possible by technological development, economic investment and the application of political capital. Going beyond the individual gene offers new diagnostic and treatment possibilities—particularly for complex and rare conditions.

Alongside these clinical opportunities, there are substantial social implications. As Martin and Dingwall (2010, 514) have noted, one of the distinguishing features of genetics (and now, genomics) is its promissory discourse which relies on a mobilisation of ‘high expectations and social anxieties’. Such can involve a reconfiguring of expectations, hopes and fears about the future (Martin and Dingwall 2010). As Parker (2012) argues, ethical problems within genetics emerge against, and need to be understood in the context of, a rich background of complex but largely day-to-day practice. This is also true for the experiences of those engaging with genomics as patients or carers. An engagement with genomics is always mediated through, and interpreted against, peoples’ own lived (and everyday) experience (Featherstone et al. 2006).

Understanding the perspectives of those who engage with genomic medicine services is (or, should be) an important facet of social scientific enquiry. Particularly, as previous research has suggested that patients’ expectations and assumptions about ethical practice may not always be the same as those of healthcare professionals (Dheensa, Fenwick, and Lucassen 2016). However, as Lewis *et al*. (2020) note, there are relatively few qualitative studies that explore the perspectives of parents who have been offered genomic sequencing to diagnose their child’s rare conditions in the UK. As a result, we are less aware of how families see the impact on their lives of the positive imagined futures presented by scientists, clinicians and policy makers. We might also be prone to reducing the experience of families down to that of travellers on the ‘diagnostic odyssey’ so often referred to in literature (Rosell *et al*. 2016). Our research aims to collate rich accounts of lived experience in order to make visible the diverse, variable and multilayered everyday lives of patients and families and how these correspond with emerging, rapidly changing, and complex fields such as genomic medicine. While the application of genomic technologies has the potential to transform patients' lives, the excitement (or, ‘genohype’ (Kakuk 2006)) around these technologies mustn't eclipse the everyday experiences of the people who live with, and care for, those with genetic conditions. As Kerr *et al*. (2021) have argued, these promissory claims are fragile and contested, particularly when set against everyday encounters.

Dealing with the uncertainty that the advent and subsequent mainstreaming of clinical genomics brings requires working in a way that empowers ‘voices at the margins so that they may help craft creative options and [create] opportunities for collective consensual decision-making that are respectful of difference’ (Baylis 2019, 175). This involves finding ways to speak ‘with’ rather than ‘for’, creating a way for the ideas and interests of stakeholder communities to rise to the fore, and hence, our interest in participatory-writing.

## Writing worlds

*Writing, as we see it, belongs at the heart of social research; it is part of research; a research method in its own right; a means of enquiry, exploration and articulation.* (Phillips and Kara (2021, 8)

Finding ways to understand, evoke and (re)present the experiences of research participants is at the heart of social scientific inquiry. Methodological plurality and creativity is increasingly celebrated as allowing for more nuanced perspectives, different modalities of knowledge and more participatory approaches to doing research with (as opposed to simply, on) participants (DeLyser and Sui 2014). The challenge, as described by Deacon (2000) is to ‘find ways to make living systems actually come alive’. Given that ‘social researchers work with participants to explore their experiences and perspectives in their own words’ (Phillips and Kara 2021, 105) there are opportunities to think more creatively about how we come to elicit, produce and obtain these words; written words, not just spoken.

As Richardson (2000, 923) argues ‘writing is also a way of 'knowing'—a method of discovery and analysis’. Explorations of using writing as a mode of inquiry have seen experimentation with different written forms (Eshun and Madge 2012; Lorimer 2018; Phillips and Kara 2021) as well as autobiographical accounts in the style of autoethnographic methods (Bochner and Ellis 2002). However, as Elizabeth (2008, 4) notes, such a use of autobiographical writing as a method remains underused and is ‘is largely confined to those sociologists who choose to write personally; participants are rarely granted a similar opportunity’. Certainly, many such examples can be found of this style of academic writing, and obviously, this is not to downplay their contributions and insights, but to draw attention to the potential ways in which such methods can be extended to engage with the knowledges of participants. There is perhaps something troubling about the way that ‘writing’ is something retained as the privileged territory of the researcher.

Participatory methods have been invoked to great success across a diverse range of settings: participatory drawing (Literat 2013), participatory photography (Prins 2010), participatory video (Kindon 2003) to name just a few. Yet there appears to remain a hesitancy to extending these principles of participation to that staple of research: writing. Many of the benefits that have been identified as resulting from social researchers using writing as method (Phillips and Kara 2021; Richardson and St. Pierre 2018) can also equally arrive from enabling those we research with to contribute illuminating understandings through the processual experience of writing. For example, in writing about writing as a method of inquiry, Richardson and St. Pierre (2018, 1428) emphasis original) reflect on the benefits of how ‘*thought happened in the writing’*. Similarly, Phillips and Kara (2021, 17) explain how ‘writing can help us to explore experiences and identify and express emotions’. Of course, such a generation of new ideas, understandings and connections through writing is not limited to those doing research, but extends to all those who might embrace and engage in the exploratory and expressive acts and processes emerging from an engagement in writing.

Asking participants to write is not an unusual method in and of itself. It is the core thing that we ask people to do when we send them qualitative questionnaires and include room for open-ended responses in our surveys. Diary methods have a rich history as an enlightening form of empirical investigation, capable of offering insights into everyday life (Latham 2003). The Mass Observation Project and Archive similarly relies on participants becoming diarists and writers, responding in a written form and recording their experiences and thoughts (McGlacken and Hobson-West 2022; Smart 2011). Yet, despite such utilisations of inscription, there still remains something of a sense of impropriety to the notion that research participants may write, rather than speak—one in conflict with, and challenging to, the privileged place which interviewing occupies within qualitative inquiry (Elizabeth 2008). In describing the Mass Observation Project, Smart (2011, 541) describes how, until more recently, sociological engagement with the written narratives produced through the Mass Observation Project has been limited ‘because the free way in which they wrote was not regarded as sufficiently rigorous for sociological analysis’. Attitudes have since changed however, to recognise the richness and depth of the narratives that many Mass Observation panellists produce (Smart 2011).

There are substantial benefits in asking participants to write about their lives (Elizabeth 2008). Using writing as a method of inquiry raises the possibility for ‘producing different knowledge and producing knowledge differently’ (St. Pierre 1997, 175). Writing creates a very different modality of representation; it allows research participants to ‘give the researcher their stories and words in an exact form’ (Deacon 2000). As Penn (2001, 50, emphasis original) argues, to write is an act of agency; ‘when we write we are no longer being done to: *we are doing*’. This can be a particularly important mechanism of representation for certain groups and narratives, particularly if writing about events where agency may have been lacking for the author. Writing thus provides a way of transferring a level of control and ownership to participants ‘in a way that traditional interviews cannot’ (Burtt 2020, 7). This could simply be about enabling participants to take the time to narrate their experiences in their own terms. This ‘giving time’ may be at odds with some forms of social science that prioritise and privilege the immediacy and synchronicity of the research interview as a strategy for overcoming anxieties about ‘premeditation’. Yet for some topics and participants, the opportunity to respond carefully and thoughtfully allows a more sensitive immersion in research. Participants can spend as long as they need to complete their written reflections, considering carefully their responses in an atmosphere less structured by the pressures of direct questioning (Burtt 2020). Participatory-writing can provide windows on subjects that would otherwise be hard to reach by virtue of their personal or sensitive nature (Phillips and Kara 2021). Writing can afford a level of safety in providing space, time and privacy to consider the framing and language in which disclosures and stories are told, navigating and articulating vulnerability and uncertainty. Participants are able to craft their voice. Such crafting may again be challenged by those concerned about the risks to the generation of ‘truthfulness’, though to assume that only through the imposition of questions on the spot during interviews are authentic and authorative accounts produced is flawed. To quote Elizabeth (2008, 14):

Writing provides researchers with access to the unique, partial and situated perspectives of our participants; we gain insight into the discourses that circulate in their social milieu and the way in which these vie for our participants' subjectivities.

Writing can enable people to overcome the effects of self-censorship, allowing self-revelatory forms of expression (Elizabeth 2008). While oral research methods are often based on the dialogue between interviewer and interviewee, writing tasks, as Elizabeth (2008) describes, can put past and present selves into dialogue with each other. Writing brings a level of flexibility that can aid progression beyond fixed questions and rigid categories and vocabularies introduced by the researcher, participants can employ their own concepts and terminology, and even, to certain extents, define the questions they wish to ask and answer (Burtt 2020). Similarly, participatory-writing as a method is less influenced by the mediating effects of the interviewer (or other focus-group participants); interjections, perceived cues, puzzled looks or requests to know that one’s narrative is making sense (Burtt 2020; Elizabeth 2008).

This is not to claim that writing produces accounts that can be universally representative or the source for generalisations about the social world, rather the impact and intention of writing as a method is to be *illustrative* (Evans 2021; Phillips and Kara 2021). By collating multiple autobiographical accounts from participants with a shared connection, social researchers are left with a ‘method through which we might investigate that more conventional social space of the collective’ (Evans 2021, 14). This in itself allows for the inclusion of multiple ‘voices’ within the final written product of research, giving a platform to participants that has the potential to be less directly mediated and subject to academic meddling.

This is not to position writing as an epistemologically ‘better’ method of inquiry than more conventional oral research practices, but rather to recognise that writing can allow the production of very different kinds of personal revelations from participants than what may be forthcoming when spoken. That creative or autobiographical written accounts may result in omissions and imperfect interpretations of the self is well recognised (Evans 2021), but such critiques can likewise be applied to the majority of social research methods. The point is to understand how different methods can allow different facets of life and self to arise to the fore. Like all methods, participatory-writing has its time and place (Phillips and Kara 2021). Elizabeth (2008) suggests that there is great value in using writing exercises in conjunction with interview or focus group discussions.

While embracing the ways that participants’ ‘crafted written work stands to provide eloquent answers to research questions and speak to research interests from original angles’ (Phillips and Kara 2021, 114) it is also important to remember that often the additional product of participatory-writing research are the conversations and reflections that occur along the way. Indeed, it is the practice and process of participating that can matter the most, rather than the outputs of words on a page—as useful and illuminating as they may be (Phillips and Kara 2021). The ethical bargain struck with participants may mean that on occasion the primary product (writing) is never seen nor shared, only the experience of producing it.

While social science may not have a long tradition of encouraging participants themselves to write as a mode of inquiry, as part of the rise of expressive and arts-based therapies there has been a renewed attention to writing as a therapeutic technique (Elizabeth 2008; Pennebaker and Chung 2007; Peterkin and Prettyman 2009; Robinson 2000). There are numerous studies that explore forms of expressive writing as a means for coping with a variety of situations (Bolton 2008; Gebler and Maercker 2007), yet few studies that take up Richardson (2000) challenge of using writing as a way of inquiring, understanding and ‘knowing’ more about such experiences—particularly in a fully participatory vein (though see Phillips and Kara (2021) for an insightful volume that catalogues recent efforts to do just this). It is perhaps such codings of writing as having ‘therapeutic’ applications which has stifled experimentation by qualitative researchers. Similarly, the assumption that writing exercises have a therapeutic component (by design, intention or as a predictable but unintended side effect) may make ethics committees cautious in their policing of such methods. Though again, the ‘research interview’ is often structured by, and experienced as, a confessional (Crowe 1998) and therapeutic opportunity (Birch and Miller 2000).

Of course, the open-endedness of writing presents challenges as well as opportunities (Phillips and Kara 2021). As Phillips and Kara (2021, 76) recognise, we write ‘within the means available to us’, with dominant discourses often infiltrating work that hopes to be creative, searching and illuminating, becoming more conventional or hegemonic. Writing obviously has the potential to be an exclusionary activity, with the possibilities for participation influenced by numerous factors such as class, gender, health and cultural differences, alongside past educational experiences, language fluency, habit, practice and even time available. Participation can be challenging and uncomfortable, and may be prefigured by pre-existing attitudes and aptitudes to the written form—and the sharing thereof. As Phillips *et al*. (2021, 54) poignantly note, ‘creative writing is not for everyone, all of the time’. Rather than acting as a participatory method, instituting writing as the method by which people take part in research can deter participation (Phillips and Kara 2021). However, McMillan and McNicol (2021, 86) suggest rather than an imposition of what writing should be, there are ways of working that can allow ‘a community’s existing ways of writing and knowing to come through’. Such can involve cultivating a sense of inclusion, safety and trust among participants (Phillips *et al.* 2021)—many of the core values of qualitative research at large, around building rapport and relationships are particularly applicable to using writing as a method of participatory inquiry.

It is important to bear in mind that writing is directly affected by the nature of the intended audience, and particularly the levels to which any audience may be imagined to have been conferred with evaluative powers (Elizabeth 2008). Similarly, and as with any form of qualitative research, accounts produced through writing are shaped by the possibilities and desires for levels of anonymity for the authors involved. Further, we must recognise that writing fixes words—and as Elizabeth (2008) notes, hence also fixing constructions of selves and narratives. Though given that most interview research involves a subsequent transcription to an often equally fixed written form, this is perhaps not too dissimilar. Indeed, the opportunity for a level of editorial oversight of one’s own words and stories is one of the benefits brought about through participatory-writing. However, while writing as a mode of inquiry can be less intrusive and less pressurised, especially when taking place in the writer’s own time and space, this also means there are also fewer opportunities for researchers to provide emotional support, reassurance or to steer questioning away from distressing topics (Burtt 2020). There are thus complex ethical considerations prior to asking participants to write, to sit and mediate on a topic, in ways that—though possibly enjoyable, comforting and self-revelatory, can also be frustrating and saddening (Elizabeth 2008; Phillips and Kara 2021).

As well as affecting those doing the writing, writing also has the potential to affect readers in ways that formal academic writing cannot (Phillips and Kara 2021). Participants’ writings can be well suited to disseminating research, particularly as the expressive nature of a participant’s writing can be seen to be ‘evoking’ emotion rather than just ‘explaining’ emotion; showing rather than telling (Andrews 2018). Participant-produced stories have an ‘enlivening’ quality, and can have a valuable role to play as communicative resources, building—and bridging—empathy (Parr 2021). Parr's work in particular demonstrates how stories can lead to changes in behaviours and styles of engagement within professional communities (Parr 2021).1

## Participatory-writing with people affected by rare genetic conditions

This research is part of a larger participatory study working creatively and collaboratively with families touched by genetic conditions to explore the experiences of patients and participants in genomic medicine and research. Our aim is to identify the overlaps and gaps between practitioner and patient accounts of ethically relevant issues as they occur in clinical genomics and find ways of supporting people to feel more comfortable when having challenging conversations. Our principal research question is whether accounts of patient experience might contribute to the preparedness of clinicians to deal with the ethical challenges of genomics practice.

It was this ambitious research question that piqued our interest in participatory writing, with the hope that such narratives might express and evoke aspects of lived experience in productive and affective ways, beyond what was possible through more conventional social-scientific registers and/or participatory practices of ‘patient engagement’. As Frank (2012) notes, equipping healthcare professionals with a sense of ‘what to listen for’ can enhance ‘professional listening’.

Co-production is at the heart of the research project. Our research is directly informed and guided by people with lived experience. We have a participatory steering group who attend project meetings, share their ideas and experiences, and contribute and comment on the design of research activities. This has allowed us to develop relationships with participants, build trust and confidence, and demonstrate our commitment to confidentiality, giving people a voice, and effecting change. Thus, we see ‘patient and public involvement’ as a constant and continual process, rather than an initial involvement at the outset of the project. Our research involves continued dialogue and input from people with lived experience of genomics and the active enrolment of their expertise, feedback and insights into the design of this project—in all its extents, including research questions, methodology, recruitment and dissemination. Our participants have acted as peer-researchers and had oversight of the writing of this article.

Conversations with members of this steering group indicated an interest in, and encouragement to, explore ways of researching using arts-based methods. Working with an author and life-writing tutor, we designed a participatory-writing programme that would encompass an hour-long online facilitated workshop on a weekly basis for 6 weeks. We were conscious of accommodating the availability of people with often complex caring responsibilities (particularly during COVID-19). Finding a mutually acceptable time to run the writing group was one of the largest challenges, although the extent to which individual participants committed to attending despite, and alongside, their commitments of caring and work (exacerbated by COVID-19 lockdowns) gave some insight into how much potential value people saw in this work. Aware of the online and disembodied nature of the groups, we sent participants a small ‘care package’ of stationery ahead of the meetings to show our appreciation and to help build a sense of occasion. We were aware that through writing (and reading others’ stories), people would be brushing up against personal and emotional topics, which could cause, or even reveal, a level of upset and anxiety. Our aim was that the writing groups might exist as a ‘safe space’ to explore these narratives. However, while we had hoped participants would find being involved in our research empowering and cathartic, we were keen to stress that our research activities did not constitute a form of ‘art therapy’. In preparing for the possibility of distress, we appointed a colleague (external to the project) with extensive pastoral experience who could be called on to support participants (and researchers) should the experience become challenging.

Initially, we recruited participants through our existing networks and relationships. A ‘purposive sampling’ technique (Sarantakos 2012) with our aim being ‘not to choose a representative sample, rather to select an illustrative one’ (Valentine 1997, 112). These were people who served as ‘patient representatives’ on ethical and governance panels relating to genomics or who were active in patient led organisations. These were people whose voices and expertise had already been sought out in different fora. Through a level of ‘snowballing’, these initial participants made suggestions for others within their advocacy networks and patient communities who they felt could contribute to—and enjoy taking part in—our research. The participant group was mixed in gender, though with a larger number of women, and ages ranging from mid-30s to early 50s. All of our participants had direct experience of genetic disease within their families, with many having a significant level of caring responsibility as a result. Our participants had experience of participating in large genomic medicine projects, such as the 100 000 Genomes Project and/or the Deciphering Developmental Disorders Study. Many of the participants we were working with had an active blogging practice, and were keen to take part in research in a way that made use of their skills, interests and communicative strengths—but also to get something out of the research themselves, in learning new ways to hone their writing practice. Others took a little more convincing, and were particularly hesitant about how doing some writing could be scientifically productive or of value.

Week by week, each session involved our professional writing tutor introducing different creative writing exercises, designed to enable both novice and experienced writers to begin to express ideas and thoughts with greater fluency. This included being introduced to, and trying out, techniques such as free-writing, narrative-distancing and writing in response to a given prompt (such as telling the story of a cherished object). At the end of each session, participants were given a creative exercise to tackle in their own time. During the introduction and after each in-session activity participants were invited to reflect directly on their experience and the content of their writing. Participants were invited, but never pressured, to share their writing with the group by reading a short excerpt. Discussion was guided by the facilitator to focus on the experience of the process of writing, and to prompt the group to notice any thematic similarities or differences in written accounts. The emphasis throughout was on building confidence in expressing lived experience.

Our first writing group—who affectionately became known as the ‘Thursday Writers’—consisted of five participants, alongside the life-writing tutor, and the first author (RG), who took a participatory role in the group, rather than solely an observational one.2 Our intent was that participants would read extracts from their writing, and hopefully share it with the research team. However, participants were quickly very vocal about wanting to share their outputs in full among the other members of the group too, and we created a secure online space where people could upload what they had written in the sessions or as part of the ‘homework’. Some participants chose to use word processors, others uploaded photos of their handwritten pages. Reflecting on the impact of reading other people’s writing and noting points of connection became a regular feature of discussions (a point we will return to later on).

Our ‘Thursday Writers’ were hugely supportive of the participatory-writing programme, and several participants supported us to recruit for a second 6-week programme with an additional seven participants (which also ended up running on a Thursday). All of the writing group sessions were recorded, with participants’ consent, which has allowed us to revisit and reflect on the types of narratives that were created and shared, alongside those which were uploaded to a ‘sharing folder’ (one for each group—it was key that the ‘safe space’ created for sharing writing was limited to the people who were in the online sessions together). Participants also consented to their written pieces being used in publications, a question which was revisited in the drafting of this article.

After each 6-week programme, we arranged reflective interviews with participants, giving everyone an opportunity to share their thoughts and experiences regarding participating in the writing groups. The written pieces that participants had produced throughout the course also served as an elicitory device during these conversations (Bagnoli 2009), allowing us to delve further into the context, meaning and emotions surrounding the participant’s writing, as well as asking them to reflect on what the piece represented or evoked that may be outside of view to others.

We have specifically chosen not to attribute interview quotes nor written pieces to individuals. This is to aid confidentiality, which was an important theme for participants (see later discussion). While we could have chosen to use pseudonyms or participant codes, these can be reductive. Through not attributing quotes or pieces of writing to individuals, we are able to demonstrate commonalities across different and diverse rare genetic conditions.

Engaging with the written pieces produced through participatory-writing involved paying close attention to the stories (and, processes of storying) that were written, aiming to better understand their impact and significance (Phillips *et al*. 2021). For us, this involved drawing on aspects of dialogical narrative analysis (DNA) (Frank 2010, 2012). Such an analytical approach involves questioning:

*What is the storyteller’s art, through which she or he represents life in the form of a story? And what form of life is reflected in such a representation, including the resources to tell particular kinds of stories, affinities with those who will listen to and understand such stories, vulnerabilities including not being able to tell an adequate story, and contests, including which version of a story trumps which other versions?* (Frank 2012, 33).

DNA involves understanding stories as artful representations of lives. It involves considering why someone might choose to tell such a story and exploring how identities are being formed and sustained by the storying process. DNA’s concern is how to speak with a research participant rather than about them, and show what is at stake in a story as a form of response. A central premise of DNA is that it does not seek to interpret stories or ‘discover truths’ that have ‘escaped the attention of the storytellers’ (Frank 2012), rather the intent is to witness stories, and enable voices to be both heard and evocative—often through positioning them into dialogue with other, but similar, diffuse voices. Thus, the purpose of a dialogical narrative analysis is not to ‘display mastery over the story, but rather to expand the listener’s openness to how much the story is saying’ (Frank 2010, 88).

## Writing everyday stories

Similarly to Phillips *et al*. (2021), our approach was not to extract stories from our participants, but enable them to recount the stories that were of importance to them. Although we were interested in the dawn of genomic medicine and the sociotechnical imaginaries involved (Mwale and Farsides 2021), we were also interested in what everyday life is like for the people who live with, and care for, those with genetic conditions, and how genomics acts to reconfigure (or, perhaps, does not) aspects of people’s lives outside of the clinic. As Prainsack, Schicktanz, and Werner-Felmayer (2014, 11) argue, genetics takes place ‘outside of the clinic as well as within. It takes place in families, patient groups, state organisations, on the Internet, and on the international market’.

The phrase ‘everyday life’ is often associated with the ‘ordinary, routine and repetitive aspects of social life that are pervasive and yet frequently overlooked and taken-for-granted’ (Pinder 2011, 223). Finding significance in the everyday and respect for the ‘mundane’ draws from a feminist commitment to understand the material conditions of people’s lived experience and practices (Hanson 1992). Attending to ‘everyday life’ allows a focus on those practices and aspects of life that are hidden by dominant narratives (Highmore 2002). An everyday perspective challenges privileging certain spaces, such as ‘the clinic’, as being the locus of how and where people experience genomic medicine, to instead explore that which exceeds these formalised encounters and overflows into other domains of life. For Lefebvre (1991, 97), everyday life is ‘what is left over’. Our participants’ writing provided us with a viewpoint of those things which are ‘left over’ from accounts of genomics. It draws our attention to the way that geneticisation (Lippman 1991) and genohype (Kakuk 2006) infiltrate everyday life, but also how, frequently, at an everyday level, these new sociotechnical regimes may have little impact. With genomics being presented as a cornucopia and salve for all manner of health and social challenges, understanding ‘what is left over’ is an important effort in making visible the inconspicuous aspects of living with rare genetic conditions. As Nicholas and Gillett (1997) argue, to begin to appreciate the bioethical issues at stake, we need to fill in the gaps that exist within our understanding, something which cannot be done without narrative insights. Similarly, reflecting on genetics practice at large, Featherstone *et al*. (2006, xii) argue that ‘a sound appreciation of everyday social reality is of profound importance for professional practice’. Thus, as Frank (2012, 36) notes, ‘to describe the world may be the most effective way to change it’.

Many of the pieces of writing that emerged from the groups touched on these sorts of everyday realities, and hidden complexities, of caring for people affected by rare genetic conditions.

With ‘My Day Begins’, I just sat down, and I was typing rather than writing freehand because I’m faster. And yeah, there it was, and I hardly had to change anything, after the first draft. I was really pleased with it, and you know I started off just trying to make it as factual as possible, as matter of fact as possible. And it wasn’t until I shared it with other people that then they went ‘Whoa’. And I went ‘Whoa? Really? This is life.’ And I thought that was very interesting.

**Figure 1:**
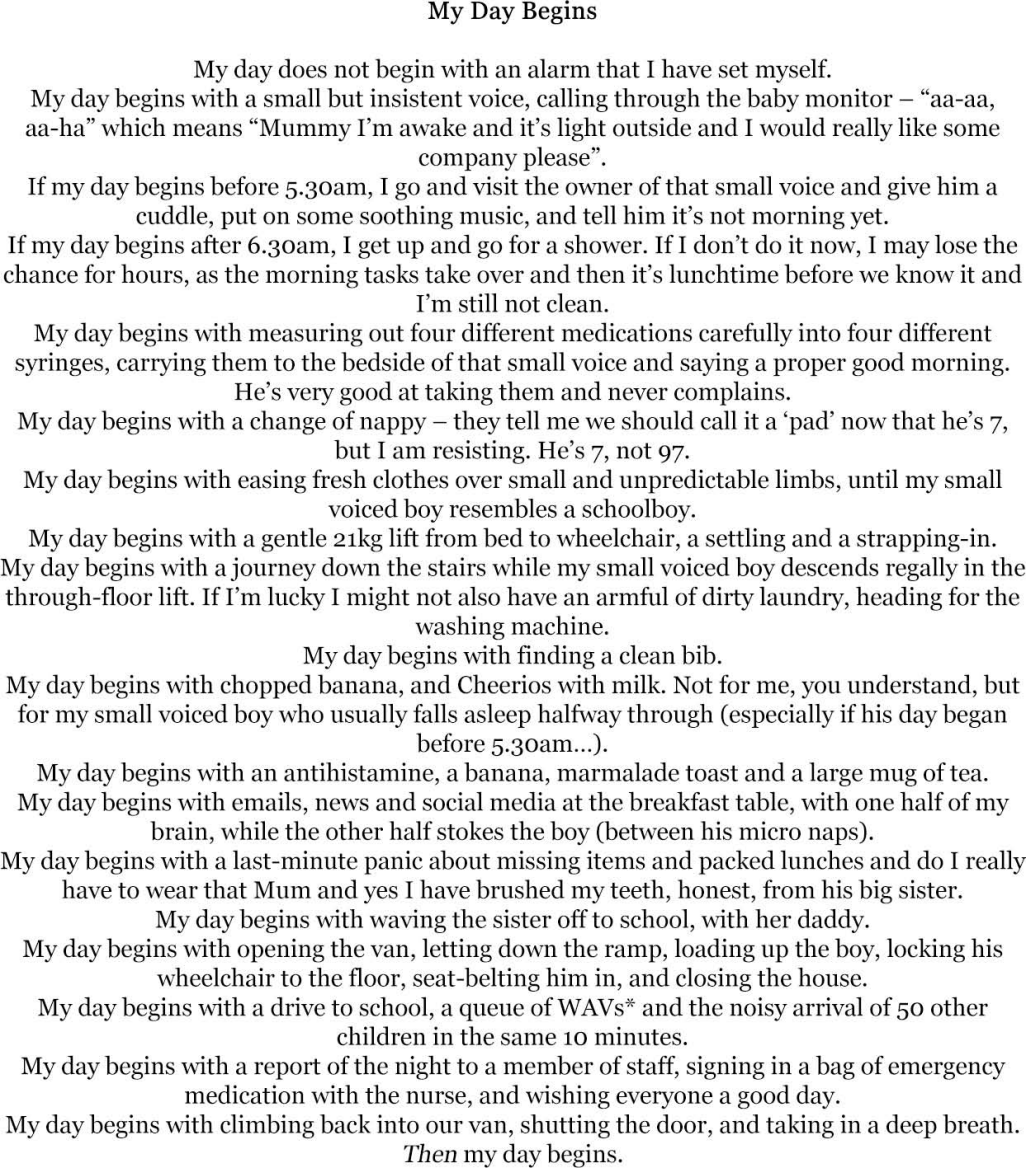
'My Day Begins'—a piece of writing from one of our writing groups. WAV, wheelchair accessible vehicle.

As one of our participants commented, writing these sorts of creative pieces allowed them to draw attention to the complexities involved in care—practical, emotional and identity-based complexities, not just medicalised complexities. As the author of ‘My Day Begins’ (figure 1) notes,

There’s stuff in there about the complexity of caring for somebody, the practical complexities, and there’s stuff about the emotional complexity of being part of a wider family unit and still having to cope. And there’s this stuff in there about having to put aside your sense of self and be a parent or a carer. And I think a lot of people who don't have caring responsibilities would never think twice about that.

**Figure 2:**
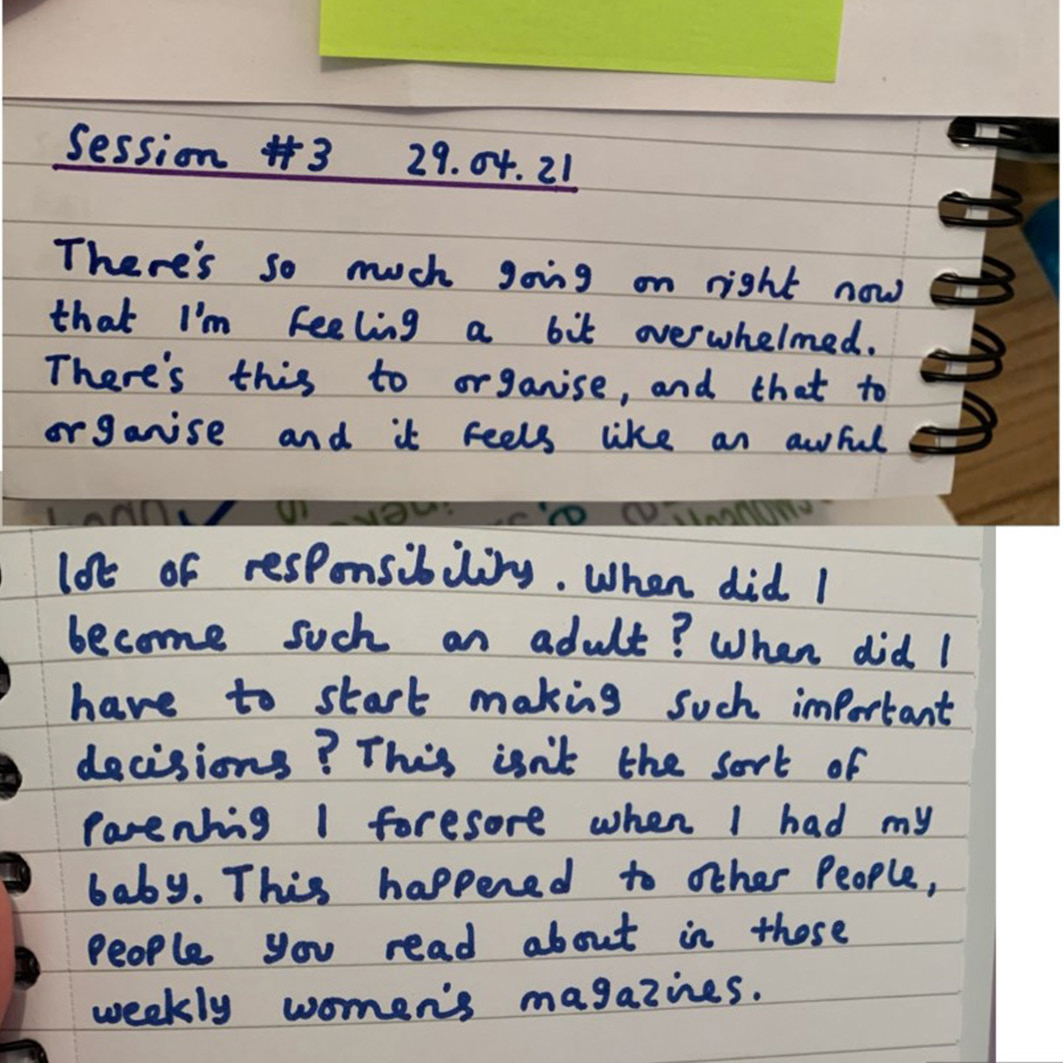
'Freewriting, Session #3'—a piece of writing from one of our writing groups.

Bury (1982, 169) noted how illness can result in ‘biographical disruptions’, where ‘the structures of everyday life and the forms of knowledge which underpin them are disrupted’. Such was certainly present in the written pieces that our participants produced. Though, rather than a singular disruption, years with no diagnosis, potential misdiagnoses and potentially having to adapt to receiving a diagnosis for a condition different to what had been expected (Dheensa, Lucassen, and Fenwick 2019), means that genomics follows multiple disruptions to both forms of knowledge and everyday life. Figure 2 exemplifies this.

Yet even in writing these representations, participants were keen to hold attention to these acts as being specifically everyday. They were aware and quite critical of the possibility for acts of interpretation to render their writing as something very different. One participant described the challenge of eliciting empathetic responses, rather than just solely sympathetic responses.

Sometimes I feel in this juxtaposition about not wanting to be personified as a superhero, because we’re not, we are just doing what the majority of parents would do if they had to do it, that is how it is.

As a route to enabling the possibility of empathetic response, many of those who took part in the participatory-writing sessions commented how their pieces perhaps captured aspects of their lives that they felt were outside the view and understanding of medical professionals. The lack of alignment between families and healthcare professionals as to what ethical practice around genomics might mean and require (Dheensa, Fenwick, and Lucassen 2016) can be produced in part by this lack of visibility and knowledge about what is important and what is experienced on an everyday level.

‘They [healthcare professionals] tend to have a close in view of it rather than a bird’s eye view of it, in a way all of that stuff and stress is invisible.’

‘I think clinic doctors perhaps don't see anything like this side of things.’

‘I’ve written quite a lot and I think other people have as well about how it feels to be a family with or without a diagnosis, rather than what the medics or what the team seems to think is important.’

**Figure 3:**
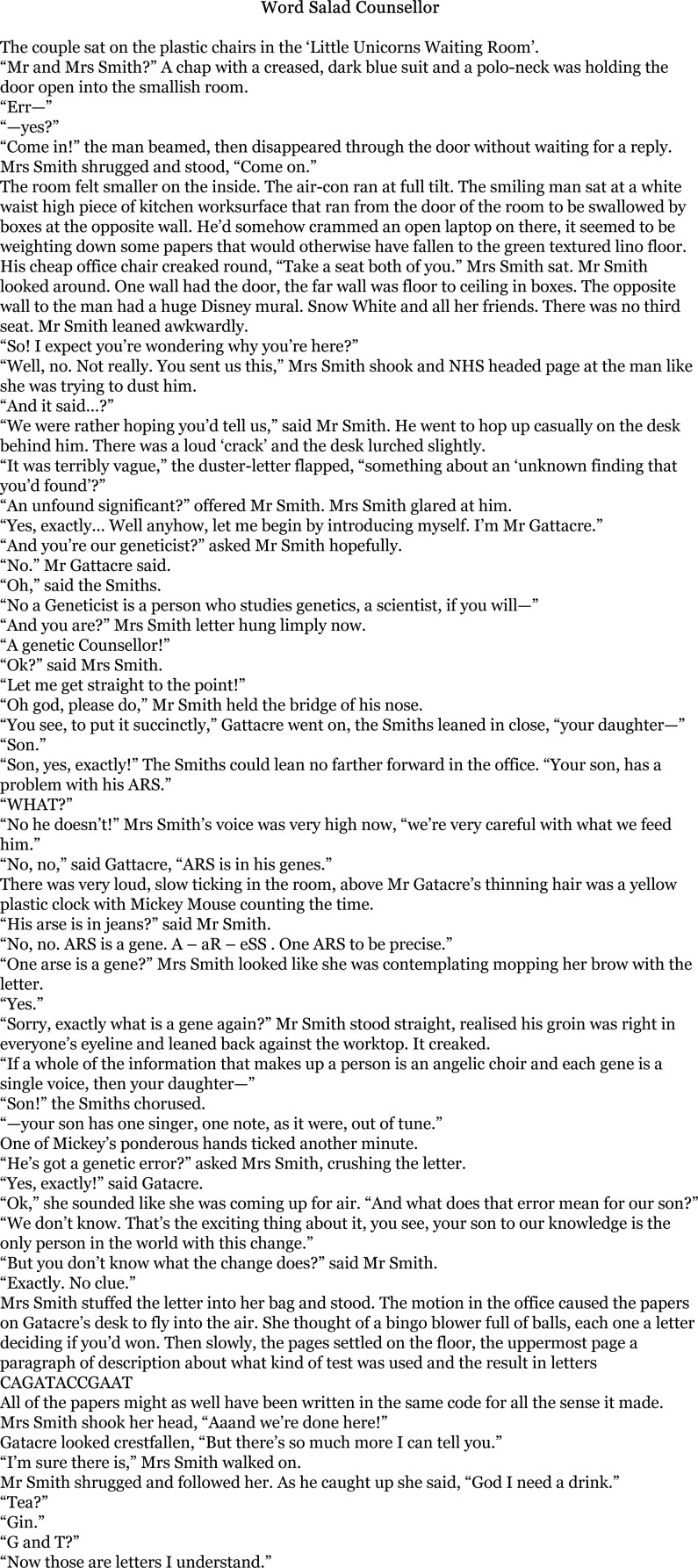
'Word Salad Counsellor'—a piece of writing from one of our writing groups.

Noting things as being exterior to more commonplace comprehensions was not always presented as disenfranchisement or critique of healthcare professionals, but rather a way of drawing attention to the multiple forms of lived expertise that parents were called on to develop and mobilise. One piece, ‘Word Salad Counsellor’ (figure 3), in particular showcased how engagements with genomic medicine required patients and parents to develop new skills and knowledges, specifically in navigating the complex scientific languages through which clinicians enact and practice care.

There is much to take away from figure 3. The use of humour to mask painful experiences; the hyper awareness of space and environment; the use of language, metaphors and similes; the lack of attention to important personal information (e.g. misgendering the child) in lieu of a focus on complex scientific information; the unfortunate use of the word ‘exciting’ when attached to what is in fact bad news for this family.

In particular, this tongue-in-cheek piece highlights how accessing genomic medicine services can require quickly learning scientific vocabulary in order to interpret clinical communications and be confident in understanding, participating and obtaining, optimum care. The challenges of the technical language surrounding genomics (and health information in general) are well established (Stuckey *et al*. 2015). The onus is frequently on the patient to acquire the expertise to interpret the information being provided—as described in the quote below from an interview with the author.

Yeah, I mean it’s amplified but it’s not amplified by very much at all. I took your world’s worst plausible genetic counsellor and went from there. The surreal-ness of it actually comes from a lot of the stuff that’s real in a way because I went back to it and thought which bits really chimed? Of course, it’s all the stuff like, ‘Oh, yes, this is a known variant of not a great deal of significance.’ All those kinds of things. To most people it sounds like Douglas Adams but it’s not, it’s just what arrives in the letter. Okay, should I worry about this? Do I need to translate it before I worry about it? What are we doing here? … I’ve rapidly built on my A level biology knowledge which was already 30 years out of date. When I learnt my genetics the human genome hadn’t been sequenced so it’s all happened in my lifetime really and it’s been a bit of a helter-skelter. In a way you’ve got to learn it … it’s all delivered in closed codes and so in order to pick any of the useful information out of that you’ve got to learn it quick.

Although not the intended purpose of the writer, the original piece of writing and the follow-up discussion provide invaluable insight into the way theory and practice come together (or do not) in the clinic. The encounter highlights the centrality of what can be described as a ‘diffusion model’ (McNeil 2013) of public engagement efforts around genomics; that is, the aspirations and follow on assumptions that groups will acquire scientific knowledge about new technologies via a ‘trickling down’ or ‘osmosis’ of information. This is slightly different from—though, entangled with—ideas that suggest a ‘deficit model’ of public engagement, that takes as its starting point a deficiency in understanding that can be solved through more or better education (Marks 2016). A diffusion approach instead assumes that those encountering a new technology will actively seek, access, comprehend and use related information. Institutionally, it is a passive (indeed, neoliberal) approach to public engagement that positions individuals as responsible for their own empowerment. In practice, the prevalence of a diffusion model of public engagement is potentially as equally problematic as the well-critiqued deficit model. Hoping that those engaging with genomics services will have acquired the confidence, knowledge and skills to equitably participate through a wider diffusion of public understanding of genomics and/or a commitment to self-education is, at best, a sticking plaster. More creative dialogical strategies for developing public engagement around genomics are still very much required (Samuel and Farsides 2018).

Participants were keen to use the groups, and their writing, as an opportunity to craft narratives and representations that resisted and challenged what they frequently felt was expected (and indeed, imposed on them) by institutions—whether the wider genomics ‘industry’, or even patient support groups. As such, participants were aware of particular types of writing that would be well received and seen to have extrinsic value, but struggled to square that with the way in which they wanted to tell their own stories and reflect those of their children.

We’re thinking more about how our children are represented, and their awareness of themselves. It’s that thing that ‘this child is disabled and they’ve had a horrible life and they’re so sick and blah-blah-blah’, and then people give you funding … And I’ve had conversations with [charity] about it before because they’d written something for a funding bid and it said ‘a lot of these children will die’, and I thought, ‘Do you really need to say that?’, and they were like, ‘Yeah, because that’s what gets people…’. But when you’re thinking about your child and how you want the world to view them and how you want them to view themselves, it’s kind of a different thing I guess.

**Figure 4:**
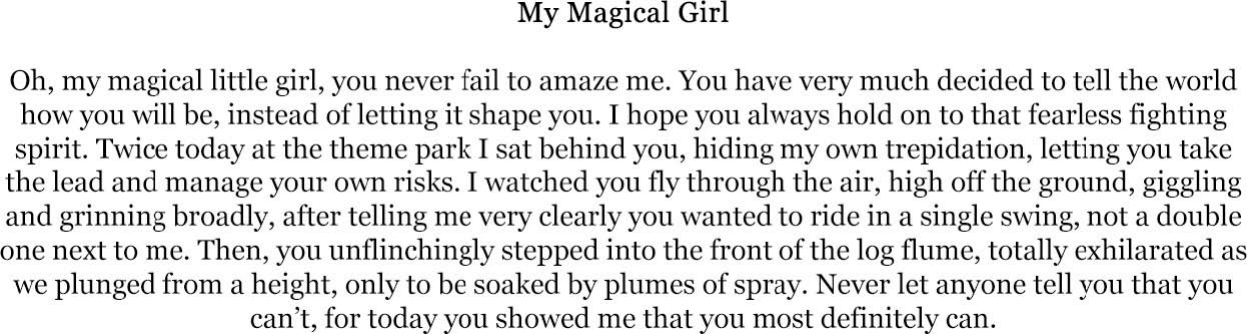
‘My Magical Girl’—a piece of writing from one of our writing groups.

Thus, many of the pieces of writing that participants created aimed to tell positive stories, ‘normal’ stories, that resisted medicalisation and politicisation, even casting it to the margins, such as in ‘My Magical Girl’ (figure 4). As one participant noted, *‘one of the things I really liked, one of the reasons that I write is to share the good stuff that happens’*. Intertwined with this, we can witness how participants are keen to reclaim and recentre certain aspects of their identities which perhaps they do not get the opportunity to voice in other (particularly, medical) contexts. As one participant reflected on their writing: ‘*I think there is that bit of still being a mum and not being a carer or a medical secretary’*. Parents of children with rare genetic conditions are often implicitly expected to become ‘expert caregivers’—something which healthcare systems rely on, though simultaneously struggle to acknowledge (Baumbusch, Mayer, and Sloan-Yip 2018).

Similarly, another participant reflecting on the writing groups explained:

We did a narrative piece that I’m just looking at now and I think that does what I like to do, which is just show some of the normal stuff around living with someone with a rare condition. Just trying to show that we do have a normal life and just showing that we do have things in common with other people, we do have things we can talk about and if you come and talk to us or read our writing, it doesn’t have to be about genetics! We’ve got other things that are in our lives and are important to us.

**Figure 5:**
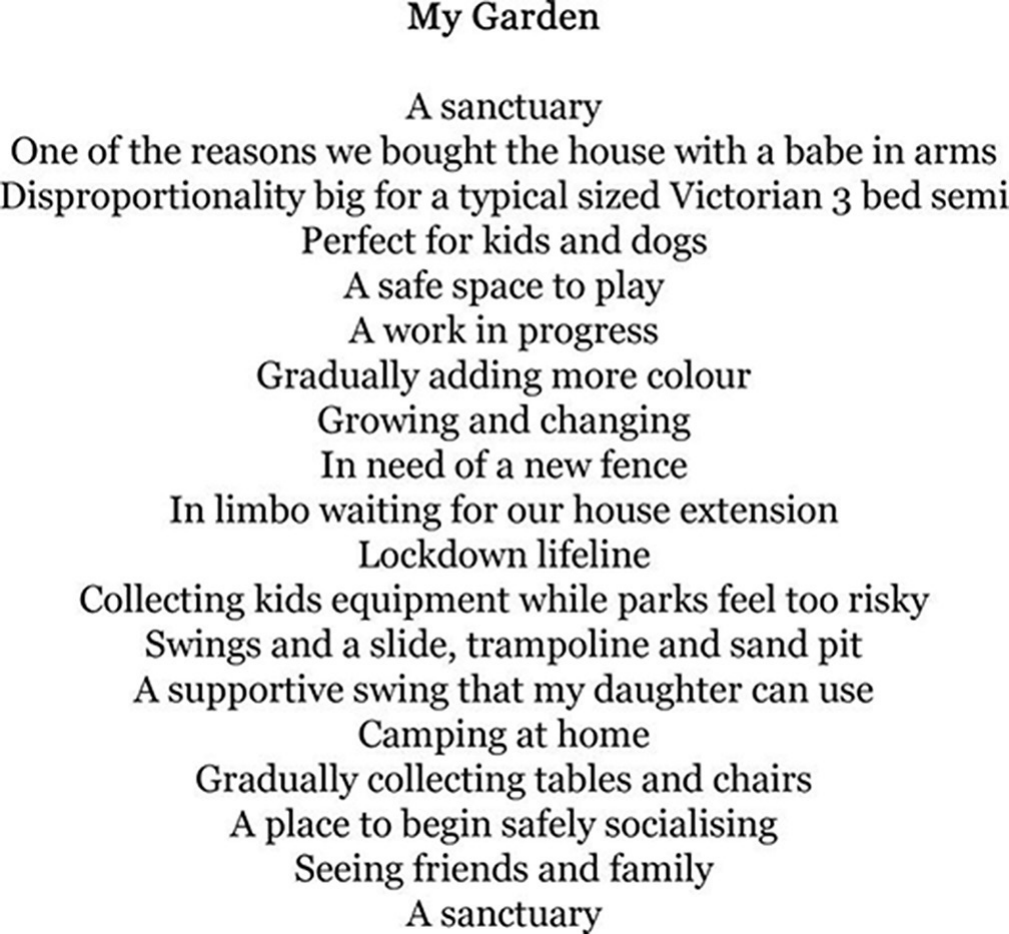
'My Garden'—a piece of writing from one of our writing groups.

At first glance, some of the written excerpts appeared to describe aspects of life quite mundane and unremarkable. However, when read through the context of rare genetic conditions, these pieces can draw attention to how such multiple aspects of everyday life are reconfigured and challenged. Indeed, one participant reflected that, *‘I don’t think I wrote particularly much about her condition per se, but then I think things leak out in whatever you’re writing about*’. While another noted how *‘no matter what we write about, you can always feel that parenting concern in the back of your mind. The inability to be completely free of that.*’. For example, the written piece ‘My Garden’ (figure 5), touches on the home adaptations and extensions often required to allow domestic spaces to become accessible, the exclusions that can be felt from public spaces lacking specialist play equipment and the vulnerabilities that a rare diagnosis can bring in pandemic times.

**Figure 6:**
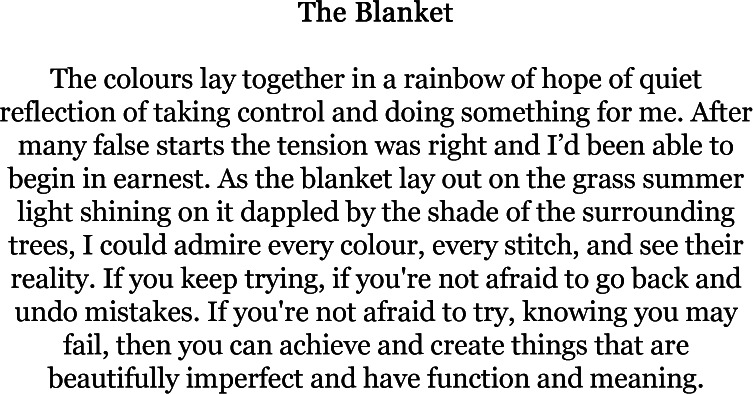
'The Blanket'—a piece of writing from one of our writing groups.

Many of the pieces written as part of our two writing groups explored some of these other things, whether descriptions of gardens, fond memories or day-to-day conversations. Not all of them were approached through the lens of rare conditions. Instead, caring responsibilities or medical paraphernalia featured as an absent-presence. Yet, many of these pieces of writing, even when not directly or explicitly mentioning rare disease, carried messages and themes that other participants took to be particularly meaningful when interpreted through their own lived experience of rare genetic conditions, such as ‘The Blanket’ (figure 6).

The Blanket was amazing because it was that kind of completionist idea, the idea that the caring and dealing with the genetic odyssey is a never-ending saga and so you never get to complete anything because you’ve got to do it again from scratch tomorrow. The blanket more than anything else kind of touched me. It’s the one I really took away with me.

Writing in this way gave participants scope and freedom to tell stories that they felt—as one participant described—‘could only happen by metaphor’. It provided a way to represent aspects of their lives and experiences that exceeded what could be conveyed in oral recollections and explanations. The blank page and freedom to write about anything was an important way of creating a space where people felt comfortable to explore different narratives, centre different identities and challenge assumptions about life with rare conditions. As one participant explained:

I was a bit worried that at the beginning, we would be invited to delve into points in our story that we felt were pivotal or particularly strong memories. And I was very glad that [the facilitator] didn't do that. She was very careful to say, this could be any of your experiences, write about any of it. And then, of course, you can choose how far you tip-toe into that or not. And that was good.

With this in mind, we want to briefly turn our attention to reflecting on the value that writing has had here, as a qualitative method, and how it has allowed us as researchers to explore the lifeworlds of families touched by genetic conditions.

## Reflecting on the value of participatory-writing for social research

Participatory-writing has enabled us to learn about many of the things that mattered to our participants. It has given us an insight into their everyday lives; the complexities, the challenges, the frustrations. Writing (and the interlinked processes of sharing and reading) has allowed our participants to voice their narratives and representations in ways that they found to be important and authentic.

The written pieces that were created—whether poem or prose—have been immensely evocative. We have included as many as possible, and in full. Particularly as, to quote Frank (2012, 36), ‘each story must be considered as a whole; methods that fragment stories serve other purposes’. Herein lies one of the challenges of participatory-writing, in that what is produced does not lend itself well to the demands and constraints of academic publishing! Though we hope to also demonstrate what is achievable even when time is short. One of the largest challenges of the method that we and our participants reflected on were discussions around privacy and confidentiality.

There’s a lot of stuff now that I have to be so careful, because of protecting the kids’ privacy. There’s stuff I’d like to talk about because it affects me, some of the conversations that we’ve had to have, I can’t put those anywhere and it’s not that I want to necessarily share that as such, it’s a difficult one in that sometimes it is just cathartic to get it out and write it out but then I’m always mindful, who is reading this? How can this come back in the future? How would that get back? How would I feel if my kids, how would they feel if this was out there?

Writing brings with it a greater sense of permeance and mobility. It produces a record in ways different to that of conversation. In our research, we always ensured that people felt comfortable to not share what they had written—and indeed, some people did not. Throughout the process we stressed the optional nature and modular approach to sharing, allowing people to choose what was shared with us as researchers, what was shared within the group, and what—if anything—could be shared more widely, for example, the pieces of writing featured in this publication. Participants assessed this issue on a piece-by-piece basis and knew that we as researchers wanted them to retain full control over everything they had written.

It was encouraging that participants reflected on how writing had, as a method, both encouraged and enabled them to detail aspects of their experience that might not have come to the fore had we relied solely on oral interviews. Writing, as one of our participants described, *‘really threw a light on those things that it’s really hard to explain under other circumstances’*. Another participant described noticing that the written medium had encouraged them to ‘dig a little bit more’ into their feelings:

[You] can twiddle with it, so like we might come out of this meeting now and I might think, ‘oh god, I wish I’d said that’. Whereas if you’ve got a week or so to actually play with it and add or take away from it or whatever, because sometimes you write something and it’s done, but then you start typing it up and you’ll just rephrase it in a different way. So, there’s that time to actually consider it.

The opportunity to creatively use metaphors, write-between-the-lines, and crucially, take the time to craft and edit narratives gave our participants the opportunity to consider how they responded, and to convey what they felt was an added level of detail. For some, it was particularly the opportunities that writing offered to make these narratives ‘more lively and interesting’ that was appealing. This liveliness is particularly important if we take seriously Vannini’s claims that social research ought to consider the *‘unique and novel ways it can reverberate with people, what social change or intellectual fascination it can inspire, what impressions it can animate, what surprises it can generate, what expectations it can violate, what new stories it can generate*’(Vannini 2015, 12). This involves recognising the performative quality of words themselves and the intersubjective means by which knowledge is co-created by writer and reader (Anderson 2014), a shift from aiming to *explain* how something might ‘feel’ to instead attempting to expressively *evoke* how something might ‘feel’.

Writing in this way has thus been a valuable method for us. But it was also an approach which our participants valued and embraced. Participants described the cathartic release of writing something and then ‘letting go of it’, something that also enabled them to have a level of distance from what was produced and represented.

I’m usually writing something because I know that I’m going to put it somewhere somebody can read it. But this, I was just writing it. I suppose in some ways that was different […] in terms of the idea of a revelation and feeling things.

Writing provided a way for participants to navigate and negotiate vulnerability on their own terms. It produced a level of solidarity and sociality among the groups too, one that acted as a counter to what one participant described as ‘the isolation and loneliness that lots of carers feel’. We’d—perhaps naively—initiated this research in the hopes of producing material that would be engaging and informative to healthcare professionals, however, it quickly became apparent that reading other people’s writing was powerful and rewarding for other families affected by rare conditions too.

I think being able to connect to what other people were saying. I know there was a piece in particular that [participant] wrote, and I felt like that could have been something that I actually could have written myself. The language that she used, the situation that it was about, it was definitely something that I thought, ‘Wow that’s my life, I could have written that.’ That was quite strange actually, it was nice though.

This overlapped with what participants felt to be another strand of value in writing about their experiences: being heard.

There’s just something about knowing that people are listening and actually giving you a really nice way to talk about things. It’s as far away from medicalised as you can get, isn’t it, just doing creative writing.

The creativity of the medium, and its differentiation from the language and communication styles associated with more clinical discourse became something that in itself had a generative potential. Participants felt enabled to claim an ownership and validity to their representations of experiences. The written form had an authority and level of definition that empowered people to write about the more-than-medical realities that constitute life with rare genetic conditions. It provided an important outlet for people to voice their narratives—often stories that they felt had no place or might even undermine their expertise. As one participant commented, ‘*nobody asks me this stuff’*. Another described how,

I think people see you swan-like gliding along, having these silly ideas about how easy you’re making it look. They don’t see any of all of the bits that are going on behind it and writing about nappies and children being resuscitated and all of that kind of thing. I suppose I feel it allows me to tell people what it’s really like.

Again, it emphasises the value of taking an everyday approach, and considering what is ‘left over’ (Lefebvre 1991), what exceeds or escapes more formalised representations of life with rare conditions, and what is absent from the genomic imaginaries and promissory discourses that are created and mobilised at a political level.

## Conclusion

In the spirit of dialogical narrative analysis, our aim has never been to ‘summarise our findings’, but ‘rather to open continuing possibilities of listening and of responding to what is heard’ (Frank 2012, 36). Stories are integral to medical care, as Nicholas and Gillett (1997, 296) argue, ‘in its representation of subjective experience, narrative gives us access to the perceptions and valuation of other human beings, and thus narrative bioethics is a means of thinking about the meaning of illness in the life of a patient and about the role of the physician in the patient-physician interaction’. The stories produced through our writing groups provide a window into the worlds of genomic medicine, the worlds outside of the clinic. They are powerful, and exist as a reminder of the wider context in which families affected by rare disease are operating—the structural, social, administrative and bureaucratic challenges which must be navigated; challenges that are compounded by one another. But also, the joys, the normality, the forgettability, the not-quite-all-consuming nature of rare conditions, and the opportunities that families find to resist a wholescale medicalisation or pathologisation of life. These stories do not provide answers or solutions. Instead, their value lies in helping to unfold the implications of experiences and illuminating what is often submerged or eclipsed by wider sociotechnical frames (Morris 2001). As Featherstone *et al*. (2006, 149) argue ‘it is vital for researchers and practitioners alike to ground their work in an understanding of everyday family practices that is sensitive to their complexities’.

We know that stories have lives, that stories travel, that stories remain memorable (Parr 2021). We hope that the excerpts we have showcased here, along with those that will be published elsewhere, might prompt greater understanding of the lived experiences of families whose lives have become entwined with the genomics agenda. Narratives can serve as a reminder of how medical practices are experienced by patients, but also how medical encounters are situated within, against and alongside everything else that happens in people’s lives (Nicholas and Gillett 1997). As Morris (2001, 55) has described, this is not a practice of thinking *about* stories, but rather a process of thinking *with* stories, ‘allowing narrative to work on us’.

## Data Availability

No data are available. Due to the highly personal, sensitive and emotional nature of the qualitative data generated, and in order to respect participant’s preferences and consent, at this stage data is not being made publicly available beyond what has been published in this article. Interested parties are welcome to contact the corresponding author for further details.

## Notes

1. There is a long tradition of using stories, narratives and writing as a way to prompt healthcare professionals to reflect on medical ethics (Jones 1999; Nelson 1997). This often takes literary sources as a starting point, though there is a growing interest in gathering stories from much more diverse places. For example, Gualtieri and Akhtar (2013) describe how blogs written by patients can offer insights and rich narratives, and provide a means to reflect on the psychosocial and emotional consequences of chronic disease. Using ‘found’ material in this way however can create complexities around consent (Hookway 2008) and thus there are opportunities to think about more equitable and participatory ways of researching and writing *with* participants.

2. RG has lived experience of a rare genetic condition themselves. The decision for RG to be present was discussed with potential participants who suggested they would be keen for them to be there.
